# NetMe 2.0: a web-based platform for extracting and modeling knowledge from biomedical literature as a labeled graph

**DOI:** 10.1093/bioinformatics/btae194

**Published:** 2024-04-10

**Authors:** Antonio Di Maria, Lorenzo Bellomo, Fabrizio Billeci, Alfio Cardillo, Salvatore Alaimo, Paolo Ferragina, Alfredo Ferro, Alfredo Pulvirenti

**Affiliations:** Department of Clinical and Experimental Medicine, University of Catania, Catania, 95125, Italy; Scuola Normale Superiore, Pisa, 56126 , Italy; Department of Computer Science, University of Catania, Catania, 95125, Italy; Department of Computer Science, University of Catania, Catania, 95125, Italy; Department of Clinical and Experimental Medicine, University of Catania, Catania, 95125, Italy; Department of Computer Science, University of Pisa, Pisa, 56126 , Italy; Department of Clinical and Experimental Medicine, University of Catania, Catania, 95125, Italy; Department of Clinical and Experimental Medicine, University of Catania, Catania, 95125, Italy

## Abstract

**Motivation:**

The rapid increase of bio-medical literature makes it harder and harder for scientists to keep pace with the discoveries on which they build their studies. Therefore, computational tools have become more widespread, among which network analysis plays a crucial role in several life-science contexts. Nevertheless, building correct and complete networks about some user-defined biomedical topics on top of the available literature is still challenging.

**Results:**

We introduce NetMe 2.0, a web-based platform that automatically extracts relevant biomedical entities and their relations from a set of input texts—i.e. in the form of full-text or abstract of PubMed Central’s papers, free texts, or PDFs uploaded by users—and models them as a BioMedical Knowledge Graph (BKG). NetMe 2.0 also implements an innovative Retrieval Augmented Generation module (Graph-RAG) that works on top of the relationships modeled by the BKG and allows the distilling of well-formed sentences that explain their content. The experimental results show that NetMe 2.0 can infer comprehensive and reliable biological networks with significant Precision–Recall metrics when compared to state-of-the-art approaches.

**Availability and implementation:**

https://netme.click/.

## 1 Introduction

Scientific investigations produce massive amounts of data collected daily in publications, databases, clinical trials, etc. In particular, in the bio-medical area, thanks to fast-track publication journals, the number of published papers has increased significantly (Ioannidis *et al.* 2023), so identifying relevant knowledge from such sources is almost impossible for a human being. In this regard, computational methods for extracting knowledge representations are a suitable tool that has supported scientists in formulating novel hypotheses and deriving new conclusions (intelligent medicine) (Qu 2022). As a result, network analysis over BioMedical Knowledge Graphs (BKG) has become a pivotal technology to uncover the fundamental biological processes underlying living organisms for precision medicine (Wu *et al.* 2023) and clinical decision support systems (Caufield *et al.* 2023); to identify new markers that indicate immune drug response in multiple cancer cohorts for immune therapies (Tagliamento *et al.* 2023, Tan *et al.* 2023); to reduce costs, time, and efficacy of clinical trials (Chen *et al.* 2022) and translational bio-medicine (Bang *et al.* 2023).

In light of these critical applications, relationship inference between biomedical entities and their representations through a BKG is of growing interest both in academia and the healthcare industry (IBM’s Watson Health, Ali Health’s medical, Google Health, etc.).

Google introduced the notion of a Knowledge Graph (KG) in May 2012 (Hogan *et al.* 2021). It is defined as a directed graph with labels on both edges and nodes. In medicine, nodes represent biomedical entities (e.g. genes, diseases). In contrast, edges (or predicates) represent relations between these entities (e.g. gene-to-disease relationships). Extracting entities and relationships from biomedical texts is challenging due to synonyms and abbreviations and is costly in data validation since it requires domain experts to check quality and accuracy (Milošević and Thielemann 2023).

BKGs can be built manually from scientific literature (high-quality but small BKGs) or automatically from ontologies, databases, or other unstructured (possibly textual) sources. In recent years, thanks to the advancements in Information Retrieval tools (Milošević and Thielemann 2023), as well as in AI/ML and natural language processing and understanding (NLP/U) (Krallinger *et al.* 2005), the research community has focused on computational approaches for extracting and modeling (possibly in the form of BKGs) valuable knowledge from several sizeable open-access article repositories [such as PubMed Central (PMC) (Beck 2010), arXiv (available at https://arxiv.org website), bioRxiv (available at https://www.biorxiv.org/ website), etc.].

The literature offers some promising approaches: (i) BioKG (Walsh *et al.* 2020); (ii) BIOS (Yu *et al.* 2022); (iii) DARLING (Karatzas *et al.* 2022); (iv) NetMe 1.0 (available at https://netme.click/ website) (Muscolino *et al.* 2022); (v) SPOKE (Morris *et al.* 2023); and (vi) Hetionet (Himmelstein and Baranzini 2015).

Finally, it is worth mentioning that other approaches build BKGs from multi-modal data, clinical trials, or specific bio-molecular interactions (Doğan *et al.* 2021, Zitnik *et al.* 2018). However, they are too specific to compare to a “generic” BKG like the one built by NetME.

Overall, the above BKGs are open source. However, only NetME 1.0 works with full texts. The others extract biomedical entities from abstracts of papers or a few paragraphs of unstructured texts. In addition, all BKGs except NetME are statically generated offline from a massive set of documents in PubMed or biomedical ontologies, thus necessitating periodic updates to keep up with new articles.

We present NetMe 2.0, an improved version of NetMe 1.0 that enables users to create on-the-fly BKGs from different sources and interact with them in a user-friendly way. NetMe 2.0 makes use of several new algorithmic technologies, including (i) OntoTagMe, a customized Wikidata-based entity linker that extends TagMe (Ferragina and Scaiella 2010) with a knowledge base of ∼3M bio-entities, (ii) a relationship-inference tool for bio-entities, developed on top of SpaCy (Honnibal and Montani 2017), (iii) an on-the-fly GraphRAG module (Cai *et al.* 2022, Li *et al.* 2022) that summarizes BKG knowledge through OpenAI, and (iv) and a set of visual tools and algorithms for network analysis working on top of the built BKG.

To assess the performance of NetMe 2.0, we conducted an experimental evaluation based on four case studies on manually curated gene–disease association (GDA) gathered from DisGeNET (Piñero *et al.* 2019) (see Section 3).

In the first case study, we evaluated NetMe 2.0’s effectiveness in extracting biomedical knowledge (in the form of entities and their relations) from a set of document IDs. Only 2 out of 46 edges were missed by NetMe 2.0, while eight misses were due to the lack of evidence in the provided PubMed IDs.

In the second case study, NetMe 2.0 was tested to extract known GDAs directly from a list of input genes. The values reported for the Recall metric were significant, ranging from 0.58 to 0.77.

In the third and fourth case studies, we compared the performance of NetMe 2.0 to that of other BKG builders. Specifically, the third case study focused on high-quality edges, while the fourth analyzed the impact of noisy edges. The raw experimental data are provided in the file “SupplementaryTables.xlsx;” the description and results of the first three Case Studies are in Sections 2.4, 2.5, and 2.6 of the Supplementary Material; while Case Study 4 is described in Section 3. Table 2 reveals that NetMe 2.0 outperforms all the other BKG builders with an absolute improvement ranging from 2% to 87%. In light of these achievements, we believe that NetMe 2.0 will help scientists to identify highly reliable relations among biomedical entities based on their (co-)occurrences and mentions in PubMed’s articles or other textual sources, thus empowering their ability to formulate novel hypotheses and derive new conclusions about their researches.

**Table 1.** Results on the BC2GM dataset (top table) and on the NCBI disease dataset (bottom table).

BC2GM dataset	Precision	Recall	F1
Baseline	0.43	0.05	0.09
OntoTagMe	0.40	0.29	0.34
PubTator	**0.81**	0.32	0.46
OntoTagMe + Pubtator	0.57	**0.43**	**0.49**

NCBI disease dataset	Precision	Recall	F1
Baseline	0.65	0.30	0.41
OntoTagMe	**0.93**	0.45	0.61
PubTator	0.85	0.49	0.62
OntoTagMe + Pubtator	0.87	**0.60**	**0.71**

In bold the best performing tool.

**Table 2.** Comparison with other BKGs—accuracy on 100 random DisGeNET associations with gene BSG.

Graph type	Tool	Correct	Edges extracted from	Web-app
Built on-the-fly with labeled and weighted edges	NetMe 1.0	63	Full-texts and abstracts	Yes
	**NetMe 2.0 (this paper)**	**87**	Full-texts and abstracts	Yes
Precomputed with labeled and weighted edges	BIOS	0	Abstract	Yes
	BioKG	48	Ontologies	No
	SPOKE	0	Ontologies	Yes
	Hetionet	2	Ontologies	Yes
Precomputed with weighted edges	Darling	85	Abstract	Yes
	BioTagMe	65	Abstract and ontologies	Yes

In bold the best performing tool.

## 2 NetMe 2.0

NetMe 2.0 is a friendly web app allowing users to visually analyze a BKG built on-the-fly from various sources, such as full texts (extracted from PubMed Central via user queries), free texts, or PDFs.

BKG construction leverages two main tasks: node/entity extraction (NE) and edge/relation extraction (RE). NE identifies biomedical entities in the input texts (e.g. genes, tumor markers, diseases, drugs, and biological processes) via OntoTagMe (see the Annotator module in Section 2.1). RE extracts the semantic relations between those entities (e.g. interactions, regulations, etc.) via the SpaCy library applied to the sentences of each input document.

NetMe 2.0 now has an enhanced front-end for better user experience and analytics. It also includes a faster rendering engine for the BKG and a richer set of functionalities for exploring the graph structure and content (i.e. shortest path computation between entities, clustering, node neighborhood exploration, connected components, BFS, DFS, betweenness centrality, PageRank). It also features a new graph-based RAG module that generates a summary text from user-selected entities (through our BKG) and their connecting paths using OpenAI.

NetMe 2.0 can be deployed through Docker, enhancing portability and scalability. Its modules and their interactions are described in the following sections. For details about the differences and upgrades with NetMe 1.0, refer to Section 2.2 of the Supplementary Material.

### 2.1 The OntoTagMe annotator module

The task of linking biomedical entities has been scarcely addressed in the literature. The most relevant tools are PubAnnotation (Kim *et al.* 2019), which annotates articles by using customizable dictionaries; PubTator (Wei *et al.* 2019, 2013), which annotates bio-concepts in PubMed abstracts and full-texts; BERN2 (Sung *et al.* 2022), which performs biomedical NER and optionally links entities to external ontologies; and (Cho *et al.* 2017), which performs Named Entity Normalization with a specific focus on plants and diseases. We decided to discard BERN2 because of its high computational requirements, operating costs, and REST API latency. PubAnnotation and (Cho *et al.* 2017) were dropped since they focus on specific annotation types for highly specialized biological tasks. Thus, we focused on PubTator due to its high-quality results, which can be effectively integrated into our entity linker.

In this paper, we developed a new *entity linker*, called OntoTagMe, that identifies sequences of *words* (or mentions) and links them to relevant *biomedical* Wikidata pages (entities). OntoTagMe extends the well-known entity linker TagMe. It focuses on linking *biomedical entities* using a subset of Wikidata that includes about 3 million biomedical pages, categorized into 15 categories (i.e. genes, tumors, diseases, drugs, and biological processes). These entities define the nodes of our BKG.

OntoTagMe’s annotations are integrated with PubTator. We assessed the reliability of the annotations on two datasets, BC2GM (Smith *et al.* 2008) for genes and NCBI Disease for diseases (Doğan *et al.* 2014), and compared the results against a baseline matching the input phrases with a biomedical ontology [DiseaseOntology (Schriml *et al.* 2018) for diseases and HGNC (Seal *et al.* 2022) for genes]. Table 1 shows their results on the two experimental datasets. OntoTagMe API is publicly available at https://sobigdata.d4science.org/web/tagme/ontotagme-api. For a detailed description of OntoTagMe, we refer the readers to Supplementary Section S1 of the Supplementary Material.

### 2.2 Network generator

The NetMe 2.0 network generator module (that improves upon NetMe 1.0) uses advanced linguistic analysis to detect verbal relations between entity pairs, representing the network’s edges and their meta-information.

**Figure 1.**
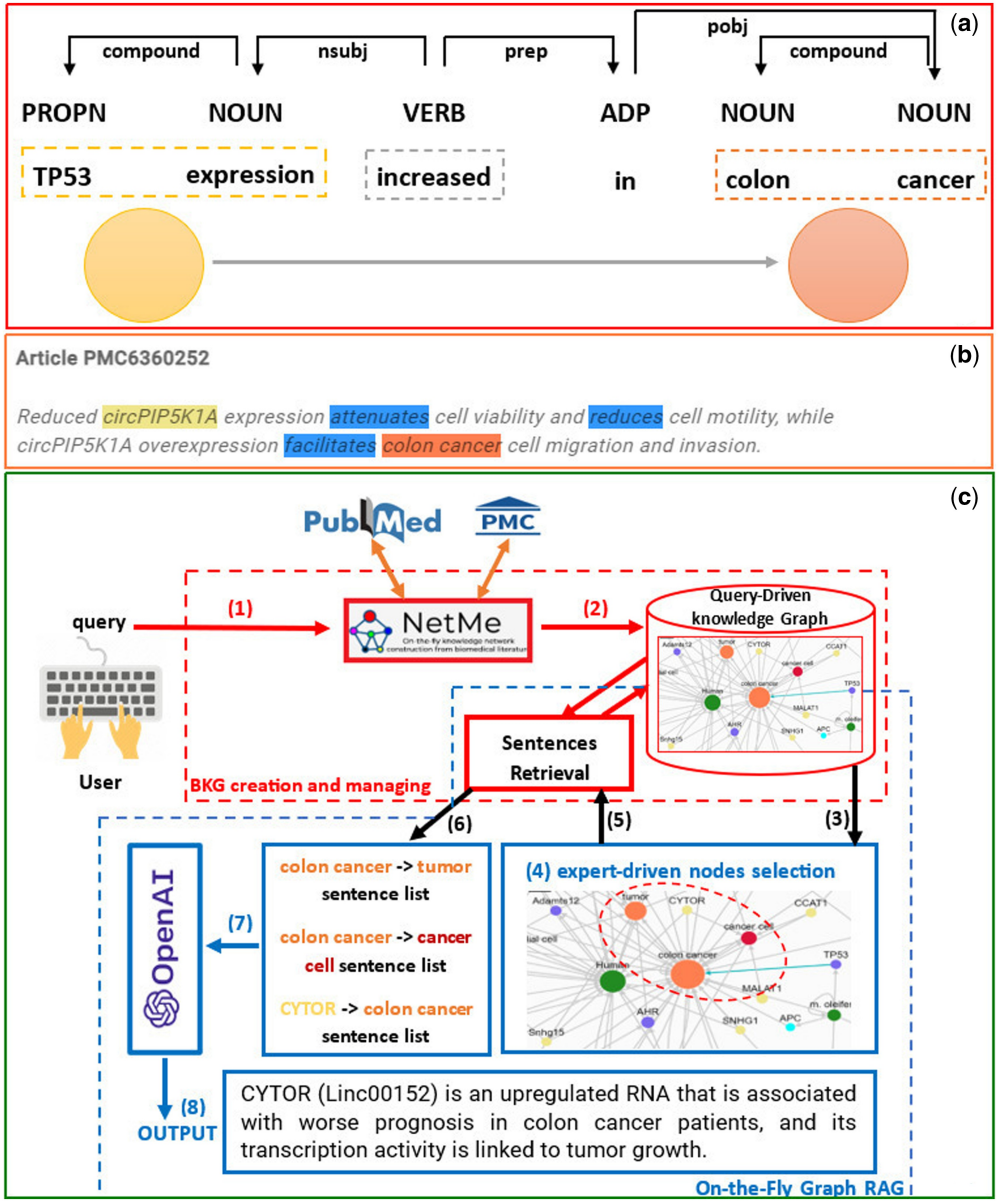
(a) Dependency-parse tree of the sentences: “TP53 expression increased in colon cancer.” (b) The three mentions “*cell viability*,” “*cell motility*,” and “*circPIP5K1A overexpression*” have not been annotated by OntoTagMe, thus the three verbs “*attenuates, reduces, facilitates*” are used to annotate the relationship between the detected mentions “*circPIP5K1A*” and “*colon cancer*.” (c) On-the-fly Graph-RAG approach. Users send biomedical queries (1) with the NetMe 2.0 GUI. Next, the knowledge graph is generated (2) by analyzing a collection of documents (from PubMed or PubMed Central) related to the user query and visualized via the GUI (3). Then, the user can select a set of nodes of interest (4), which are passed to the Sentences Retrieval module (5) to extract some phrases associated with the paths connecting such entities (6). These sentences are then transmitted to OpenAI (7) to generate a summarized text (8) explaining the (biomedical) relationships among those entities.

Specifically, the network generator module splits each document into sentences through the spaCy pipeline. Each sentence is then tokenized into words and tagged with their part of speech (PoS). We keep only the biomedical words. Next, SpaCy builds the dependency-parse tree of each sentence to extract the syntactic relationships between its tokens. This dependency-parse tree is also used to get labeled relationships (edges) between biomedical entities. When we have just one action between the source and target node, the edge label corresponds to that action. For example, in Fig. 1a, the edge from “TP53 expression” to “colon cancer” is labeled with “increased.” Conversely, if the number of actions is more than one (see the example in Fig. 1b), the edge label is formed by concatenating such actions. We score each edge e=(a,b), connecting the entities *a* and *b*, with three values: TF-IDF, bio, and ambiguity. The TF-IDF measures the relevance of an edge *e* in the $N_{e}$ input documents. The *bio*-parameter is the *normalized edit distance* between the edge label and a set of biological verb forms (listed in Supplementary Table S3). The *ambiguity* is the number of actions that compose the edge label. Indeed, the presence of many actions annotating (a,b) could be due to missing annotations by OntoTagMe (see Fig. 1b). Therefore, (a,b) could be a false positive. To deal with these, we penalize the edge weight based on the number of actions. A final score for the nodes is computed as their *personalized PageRank* (Page *et al.* 1999) (see step 6 in Section 2.1 of the Supplementary Material), in which NetMe considers the nodes in the query as teleporting nodes. Finally, NetMe 2.0 shows the BKG by our front-end GUI developed with AngularJS and CytoscapeJS (see Supplementary Fig. S4). Additionally, it allows users to report annotation errors or missing entities (see Supplementary Fig. S9), which will be used to periodically update the OntoTagMe knowledge base after manual checking. All the details of the GUI are available in Section 2.3 of the Supplementary Material.

### 2.3 Retrieval augmented generation based on our biomedical knowledge graph

Products built on Large Language Models (LLMs), such as OpenAI’s ChatGPT (https://openai.com/), generate human-like text by predicting the likelihood of a term given the preceding ones via transformer-based architectures. However, current LLMs are “frozen in time” since (i) frequently updating their training datasets is impossible, (ii) lack domain-specific knowledge, (iii) are trained for generalized tasks [see, e.g. ChatGPT (OpenAI 2023) or LLama (Touvron *et al.* 2023)], (iv) generate responses based on patterns learned during training, and (v) cannot actively retrieve specific information.

Retrieval Augmented Generation (RAG) enhances LLM capabilities by combining generative pre-trained models with information retrieval systems. It fetches up-to-date context-specific data from an external database, making them available to a generalized LLM and the user query, reducing the likelihood of hallucinations. The result is a boost in the performance and accuracy of GenAI applications, which can return more context-aware, precise, and informed responses.

However, for complex queries, RAG may retrieve ambiguous or uncertain sentences. So researchers proposed combining RAG with Knowledge Graphs [aka, Graph-RAG, see, e.g. Sun *et al.* (2023)] to understand the intent of complex queries better.

In this context, the BKG built by NetMe is a perfect candidate for designing such a Graph-RAG application. We call this novel approach *on-the-fly Graph-RAG* and refer the reader to Fig. 1c to illustrate its structure.

Specifically, a user selects two or more nodes in the constructed network via a simple search box (see Supplementary Fig. S12). Then, NetMe 2.0 computes all paths connecting all pairs of selected nodes and evaluates their score as the average of their edge weights [(TF-IDF * bio)/ambiguity]. Finally, the most relevant paths with their sentences are selected to maximize coverage of the topics of the selected entities.

Such sentences are then sent to OpenAI GPT-3.5 [the “gpt-3.5-turbo-instruct” model (Roumeliotis and Tselikas 2023)] to generate a well-formed text explaining the relationships among the user-selected entities. The temperature parameter has been set to 0 to mitigate “creativity” and “stochasticity” in the summarized text. In addition, NetMe 2.0 introduces proper citations into the summarized text (see Supplementary Fig. S13) so the user can directly check their significance, thus possibly detecting GPT hallucinations.

## 3 Experimental evaluation

To assess the quality of the BKG built by NetMe 2.0, we conducted an experimental evaluation based on manually curated GDA gathered from DisGeNET.

Therefore, we designed four case studies to assess NetMe 2.0 accuracy. The first evaluation involves queries *guided* by paper IDs specified by the user, thus evaluating the effectiveness of knowledge extraction from a set of papers via the comparison with the GDAs present in them and annotated in DisGeNET. The second case study queries NetMe 2.0 with gene names, like in PubMed, to evaluate its ability to infer the same GDAs found by experts in DisGeNET and compares NetMe 2.0 with DARLING and BioTagMe in terms of Precision and Recall metrics. Finally, our third and fourth case studies compare NetMe 2.0 annotations against those found by other state-of-the-art BKG builders. Specifically, the third case study focuses on high-quality edges, while the fourth one analyzes the impact of noisy edges. We measure the retrieval quality of NetMe 2.0 by the Recall metrics since it may detect GDAs that are not in the manually curated set, but these should not necessarily be classified as false positives since they could be yet good annotations. For example, the gene APP has 485 links on DisGeNET, but only 76 are manually curated. The raw experimental data are provided in the file “Supplementary Tables.xlsx;” the description and results of the first three Case Studies are in Sections 2.4–2.6 of the Supplementary Material. Below, we describe Case Study 4.

### 3.1 Evaluation with other biomedical knowledge graphs

We evaluated the recall of existing BKGs against NetMe 2.0.

Since we have three types of BKG builder algorithms, on-the-fly, offline, and ontology-based (see Table 2), we tested the systems to identify DisGeNET GDAs for the gene BSG. First, we extracted 100 random GDAs, each with a list of supporting PubMed articles. For NetMe 1.0 and NetMe 2.0 we counted the amount of correctly identified GDAs on the BKGs built on such abstracts. To test DARLING, we built a network from all PubMed abstracts containing “BSG” and checked how many GDAs were correctly retrieved (no filtering). Finally, we counted how many GDAs were present in all the other BKGs.

Table 2 shows that NetME 2.0 outperformed all the other BKGs in identifying GDAs. First, it significantly improved accuracy (+38%) compared to NetME 1.0, obtaining 24 additional correct relations. It yields better results than other BKGs, as they miss many GDAs. Finally, it obtained more reliable results than DARLING, even if the latter has a performance close to NetMe 2.0. In fact, DARLING links nodes using their co-occurrences in abstracts without considering their role and actions.

## 4 Conclusion

This paper presents NetMe 2.0, an easy-to-use platform for inferring BKGs from PubMed and PMC papers, free text, or PDFs. It uses OntoTagMe, a customized version of TagMe, and a syntactic analysis module based on the Python SpaCy libraries. Additionally, it includes an innovative module that enables on-the-fly Graph-RAG inference by summarizing human-like text on selected sentences from the BKGs. Our results show that NetMe 2.0 accurately extracts reliable and complete BKGs when documents cover the searched topic in-depth.

In future work, we plan on integrating the UMLS Metathesaurus (Yip *et al.* 2019) into OntoTagMe to possibly improve the quality of the annotation process. Additionally, we foresee the construction of a full-text bio-KG derived from the whole set of open-access full-text papers present in PubMed Central by extending the algorithmic architecture of NetMe to scale to million (full-text) papers and beyond.

## Supplementary Material

btae194_Supplementary_Data
