# Supplementary material for: NetMe 2.0: a web-based platform for extracting and modeling knowledge from biomedical literature as a labeled graph
Source: Bioinformatics. 2024 Apr 10;40(5):btae194. doi: 10.1093/bioinformatics/btae194 (PMC11074003; doi:10.1093/bioinformatics/btae194)
Supplement: btae194_Supplementary_Data [file btae194_supplementary_data.pdf]

Bioinformatics
doi.10.1093/bioinformatics/xxxxxx
Advance Access Publication Date: Day Month Year
Manuscript Category

OXFORD

NetMe 2.0

A web-based platform for extracting and modeling knowledge as labeled graphs from biomedical literature

Antonio Di Maria [1,*], Lorenzo Bellomo [3], Fabrizio Billeci [2], Alfio Cardillo [2], Salvatore Alaimo [1], Paolo Ferragina [4], Alfredo Ferro [1], and Alfredo Pulvirenti [1,*]

[1] Department of Clinical and Experimental Medicine, University of Catania, Catania, Italy.
[2] Department of Computer Science, University of Catania, Catania, Italy.
[3] Scuola Normale Superiore, Pisa, Italy.
[4] Department of Computer Science, University of Pisa, Pisa, Italy.

*To whom correspondence should be addressed.

1 Details on OntoTagMe

This section provides implementation details of OntoTagMe, along with a more thorough description of the results achieved by combining OntoTagMe and PubTator.

1.1 Creation of the knowledge base

We have chosen Wikidata as the biomedical knowledge base of OntoTagMe since it offers a tabular-like view of biomedical entities with a minimal but straightforward description. Information is expressed through a set of pre-defined properties, ranging from generic ones (e.g., *"part of"*) to specific ones (e.g., *"gene substitution association with"*). Aliases are provided directly as a field of Wikidata pages.

From Wikidata, we created a set of *Wikipedia-like* files that we used to specialize TagMe to biomedical entities. More specifically, we need:

- a *Category* file, which contains a set of relevant macro-categories identifying the main types of bioentities (shown in Table 2);
- a *Page* file, which contains all Wikidata pages that have at least one of the biological categories. We identify the category using their properties P31 ("instance of"), P361 ("part of"), or P279 ("subclass of");
- a *Redirect* file, which contains the aliases of every selected page;
- a *Pagelinks* file, which contains the set of edges (A, B) indicating that the Wikidata page A contains a link to the Wikidata page B;
- a *External IDs* file, which contains a table linking each page to their ID in an external ontology (e.g., NCBI IDs for genes). This file is crucial for the OntoTagMe and PubTator integration, as shown in Section 1.3.

We report some statistics of the extracted Knowledge Base in Table 1, while Table 2 shows the number of entities for each "macro-category".

Table 1. Statistics on the subset of Wikidata restricted to its biomedical-related pages

Set	Size	Set	Size
Pages	3 545 733	Avg. # catg per page	4.87
Aliases	4 635 736	Page links	8 012 196
Categories	17 279 837	External IDs	1 670 352

Table 2. List of biomedical macro-categories and their size, expressed in number of modeled entities

Macro-Category	Number	Macro-Category	Number
gene	4 406 910	cell type	10 245
gene variant	35	cell	1 053
biological pathway	7 178	symptom	4 454
cellular component	14 039	species	136
compound	2 700 398	physio. condition	263
chemical entity	10 715	disease	98 236
enzyme	49 880	drug	23 569
protein	3 385 962		

1.2 Annotation process

OntoTagMe is built on the publicly available code for TagMe[1], specialized for indexing all metadata (such as categories, WikiData URL) that refers to the ~ 3 million biomedical Wikidata pages.

Our decision to use TagMe was based on its flexibility, customizability, publicly available code, and efficiency.

The annotation process is then identical to TagMe. It consists of two main phases: Mention Selection (i.e., detect parts of input text that potentially refer to an entity) and Mention Disambiguation (i.e.,

[1] https://github.com/gammaliu/tagme

© The Author 2015. Published by Oxford University Press. All rights reserved. For permissions, please e-mail: journals.permissions@oup.com

for each mention, choose the candidate entity that best explains it via a collective agreement vote among the candidate entities of the other mentions occurring close to the one being disambiguated). The process incurs some "noise", tackled by a proper algorithm. More specifically, NetMe 2.0 filters out from the annotations the Wikidata pages whose title includes a common word, such as "case", "patch", or "line", which usually come as aliases of various genes.

Additionally, NetMe 2.0 filters out the mentions that match all of the following constraints: (i) composed of just one word, (ii) have no numerical characters, (iii) have no special characters, (iv) they are formed either by all lowercase or have only the first letter capitalized. NetMe 2.0 then stems all the mentions and checks whether any of them match. Then, it takes all the matches and checks whether their macro-category is equal. In that case, NetMe 2.0 "uniforms" the annotations by adopting as name and list of categories the ones provided by OntoTagMe. For example, say OntoTagMe and PubTator find as a mention "cancer" and "cancers" respectively, and both of them have "disease" as an annotated biomedical category. In this case, NetMe 2.0 can change the metadata associated with the annotation to the one assigned by OntoTagMe because their stemmed mentions are equal and their category is the same (disease).

At the end of the annotation process, OntoTagMe yields a list of entities (with their associated metadata and mentions) in JSON format. Each entry in the list of JSON annotations is formatted as follows:

- *WID*: internal numerical identifier.
- *mention*: substring of the input text where the entity was found.
- *start_pos*: index of the starting character of the mention in the input text.
- *end_pos*: index of the ending character of the mention in the input text.
- *Word*: title of the Wikidata page associated with the mention.
- *categories*: list of categories associated with the Wikidata page, comma separated.

1.3 Integrating PubTator

OntoTagMe has been designed to be easily interoperable with other Bio-NER tools. Specifically, we investigated the integration of PubTator and BERN2, two effective linkers of biomedical entities, exploiting some external ontologies. Eventually, we chose PubTator over BERN2 since PubTator is a fast and effective bio-NER tool that annotates PubMed abstracts and full texts by using advanced deep-learning techniques to link entities to external ontologies (i.e., NCBI Entrez gene ID for genes) and biomedical categories (i.e., genes/proteins, genetic variants, diseases, chemicals, cell lines, and species).

Using two linkers may lead to ambiguous annotations, yielding different entities for the same mention. To address these cases, we apply the following disambiguation policy:

- PubTator and OntoTagMe find an annotation for the same mention, then only OntoTagMe's is kept since it offers a broader categorization.
- PubTator annotates a mention that OntoTagMe missed. In that case, two possible possibilities may arise: (i) if PubTator provides an external ID that refers to a Wikidata page, the entity takes the name of that page, and its categories are enriched with those found in Wikidata; (ii) otherwise, the PubTator annotation is used with no link or enrichment to external entities.

1.4 On OntoTagMe performance

OntoTagMe is efficient and effective in the biomedical entity linking task. Indeed, unlike most state-of-the-art tools that need large language models (LLM) and significant computational power, OntoTagMe takes about 24 seconds to annotate $15,000$ short phrases on a commodity machine of the SoBigData infrastructure equipped with $5GB$ of RAM, yielding a significant impact on the yearly cost of operation.

We tested OntoTagMe on two datasets to measure its performance on gene and disease annotations:

- *BC2GM*: Gene mention dataset. It has $15\,000$ short phrases with $18\,265$ gene mentions. We used this dataset since genes are critical in biomedical research, and OntoTagMe performs the worst on genes, showing the effectiveness of OntoTagMe and PubTator integration;
- *NCBI disease*: Disease mention dataset. It has 100 short phrases with 961 disease mentions.

We compared OntoTagMe against a baseline that matches the input phrases with a biomedical ontology: DiseaseOntology for NCBI-Disease and HGNC for BC2GM. We compared the baseline against (i) OntoTagMe, (ii) PubTator (using the public REST API), and (iii) their integration. Table 1 of the main paper shows the results on the two experimental datasets. On the BC2GM dataset, we notice that the baseline gets an F1-score less than 10%, and OntoTagMe achieves an improved F1 of 34%, lower than PubTator's 46%. PubTator achieves a significant 81% precision on genes, but its conservative detection approach lowers the recall to 32%. Rather significant is the performance achieved by the combination OntoTagMe+PubTator that yields the best recall ($+11\%$ absolute w.r.t. PubTator) and F1-score ($+3\%$ absolute w.r.t. PubTator). However, all these metrics are lower than 50%, likely due to the sheer amount of inconsistencies in gene naming. For example, the gene IGKV1D-39 has alias "O2", or polysemic gene names like "displaced"[2] or "attenuated"[3]). On the NCBI dataset, OntoTagMe is the best performer for the *precision*, but the combination of PubTator and OntoTagMe does best regarding F1 and recall.

2 Details on NetMe 2.0

Figure 1 shows the architecture of the new system. In this section, we thoroughly explore some details about the edge inference module of NetMe 2.0, the new and improved Graphical User Interface (GUI) (Section 2.3, and comments on the experimental results obtained in Case Studies 1, 2, and 3 (see Sections 2.4, 2.5, and 2.6 respectively).

The extraction of relationships (the edges of the Knowledge Graph) between bioentities is an important task for modeling information of biomedical texts. Various approaches have been proposed in the literature to address this task:

- In (Zitnik *et al.* (2018) and Szklarczyk *et al.* (2018)), an edge is extracted between two entities if they co-occur in a sentence.
- In (Carvalho-Silva *et al.*, 2018), an edge is extracted between two entities if they co-occur in a sentence and they are "connected" by a verb predicate, which is then used to label that edge.
- In (Krallinger *et al.* (2020), and Krallinger *et al.* (2017)), an edge is extracted as in the previous item, with the additional feature of normalizing its predicate into a well-defined but very restricted set of types (such as is-a, part-of, equal).

NetME 2.0 extracts edges based on a sophisticated pipeline described in the following section.

2.1 NetMe's network construction

NetMe's seven-step pipeline to construct the Biomedical Knowledge Graph (BKG) can be detailed as follows.

[2] https://www.wikidata.org/wiki/Q29726548
[3] https://www.wikidata.org/wiki/Q29732195

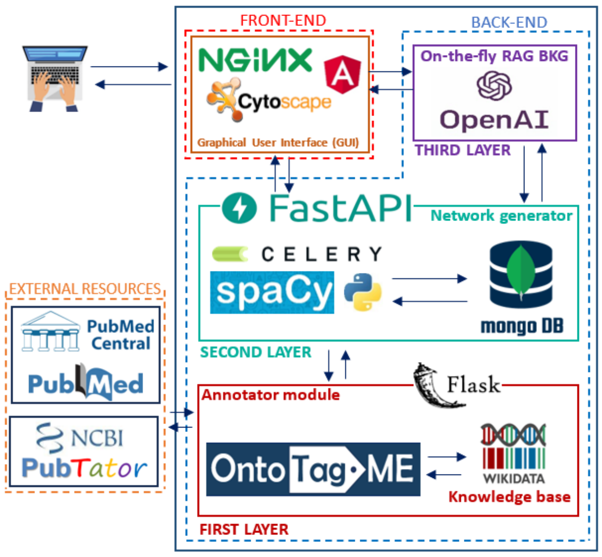

Fig. 1: A graphical description of the NetMe 2.0's architecture, consisting of a front-end and a back-end. The back-end comprises three main software layers: the Annotator module, the Network generator module, and the on-the-fly RAG Biomedical Knowledge Graph module

Step 1 (sentence splitting) : Each input document d_i is divided into a sentence sequence $(sent_1, ..., sent_M)$ through the SpaCy pipeline. Such a pipeline employs a set of rules based on punctuation and statistical language models extracted from the Spacy corpus.

Step 2 (node tagging and extraction) : Each sentence is split into tokens (words) and tagged with their part of speech (PoS), such as noun (NN) or verb (VBD). Then, each token is lemmatized to its base form to reduce linguistic variants. Finally, the non-verb tokens are filtered with OntoTagMe annotation to keep only the biomedical ones.

Step 3 (dependency parsing) : Then, SpaCy builds the dependency-parse tree of each input sentence for extracting the syntactic relationships between its tokens. Irrelevant PoS (i.e., stop-words, URLs) are also filtered out. More details in Figure 2.

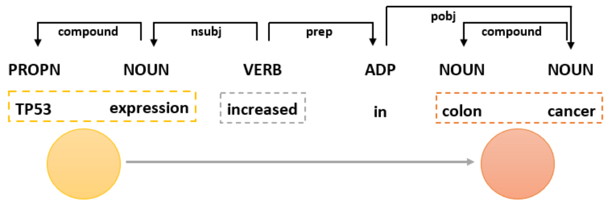

Fig. 2: Dependency-parse tree of the sentences: “TP53 expression increased in colon cancer”

Step 4 (edge labeling and extraction) : The dependency-parse tree is also used to extract labeled relationships (edges) between biomedical entities. When we have just one action between the source and target node, the edge label corresponds to that action. For example, in Figure 2, we label the edge from “TP53 expression” to “colon cancer” with the label “increased” because they are connected with that verb in the tree. Conversely, if the number of actions is more than one (see the example in Figure 3), the edge label is formed by concatenating such actions.

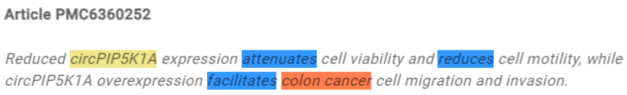

Fig. 3: The figure shows that the three mentions *“cell viability”*, *“cell motility”*, and *“circPIP5K1A overexpression”* have not been annotated by OntoTagMe, thus the three verbs *“attenuates, reduces, facilitates”* are used to annotate the relationship between the detected mentions *“circPIP5K1A”* and *“colon cancer”*

Step 5 (edge scoring) : We score each edge $e = (a, b)$, connecting the entities a and b, with three values: TF-IDF, bio, and ambiguity. The TF-IDF measures how relevant an edge e is in the N_e input documents. The *bio*-parameter measures the *normalized edit distance* between the edge label and a set of biological verb forms (listed in Table 3). The *ambiguity* is computed from the number of actions composing the edge label. The existence of many actions annotating (a, b) implies the possible lack of entities annotated by OntoTagMe (see Figure 3) between a and b. Therefore, (a, b) could be a false positive. To deal with these, we penalize the edge weight based on the number of actions: in the example of Figure 2, the ambiguity is 1. In the example of Figure 3, it is 3.

The GUI combines TF-IDF and ambiguity parameters into one score (TF-IDF / ambiguity), called *Min weight*.

Table 3. List of biological verb forms

Verb Forms		
activate	downregulate	reduce
affect	enhance	regulate
associates	express	release
block	find	reveal
cause	inactivate	stimulate
contain	increase	trigger
control	induce	ubiquitination
decrease	interacts	upregulates
detect	overexpress	
display	produce	

Step 6 (node scoring) : After gathering nodes and edges, NetMe computes a score for each node using its personalized PageRank. The teleportation step is biased towards the nodes (entities) specified by the user's query.

Step 7 (network construction and visualization) : Finally, NetMe 2.0 shows the BKG by our front-end GUI developed in AngularJS + CytoscapeJS (see Figure 4). Additionally, it allows users to report any annotation errors or missing entities by the suitable panel (see Figure 9), which will be used to periodically update the OntoTagMe knowledge base after our manual check.

2.2 Differences with NetMe 1.0

Here we highlight the main differences between the NetMe versions 1.0 and 2.0. First, OntoTagMe has enhanced TagMe by using a specialized set of

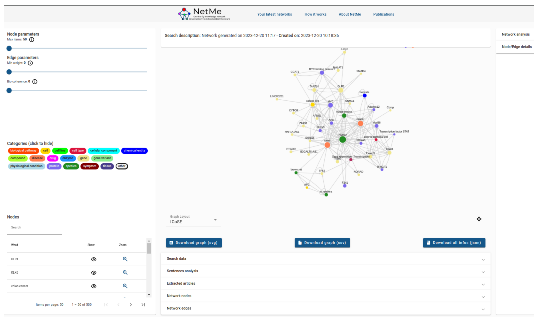

Fig. 4: The Graphical User Interface (GUI) of our tool NetMe 2.0 on an example query

3 million biomedical Wikidata pages and integrating PubTator annotation results. Thanks to that, OntoTagMe has improved the annotation quality, as shown in Table 2 of the main paper.

The second difference involves edge inference, labeling, and weighting steps. Both versions of NetMe use SpaCy for the linguistic analysis of texts, but NetMe 1.0 uses a verb-centric approach to infer entity relationships, which results in many redundancies. Given a verb in the processed sentence, NetMe 1.0 derives edges by computing all combinations among its children over the dependency tree. Conversely, NetMe 2.0 traverses the dependency-parsing tree using PoS to link entities to their verbs. For example, in Figure 3 of the main paper, NetMe 1.0 generates three edges (one for each verb) between “circPIP5K1A” and “colon cancer”, which might not be semantically correct since the action between the two nodes is mediated by elements not detected by NetMe. NetMe 2.0, instead, generates one single edge with a concatenation of verbs as a label and a score weighted according to the list length, thus keeping the indirect connection between the two nodes while highlighting that it has originated from an indirect connection.

Finally, NetMe 2.0 has an improved implementation compared to NetMe 1.0. It features a Rest API developed using Python FastAPI[4] (not available in NetMe 1.0), a redesigned front-end using Angular JS, and a back-end developed using Python Celery library[5]. Celery has been used for horizontal and vertical scaling, and Docker facilitates its deployment. Our novel platform has a sophisticated GUI equipped with network visualization and analysis tools, including the innovative on-the-fly Graph-RAG module.

2.3 GUI

Figure 5 illustrates the interface to interactively generate a BKG via PubMed query (see Figure 5A), free text (see Figure 5B), or by uploading PDF documents (see Figure 5C), which constitutes the input to the annotation and network construction procedure.

The network construction procedure produces a directed labeled graph in JSON format, which is displayed by the GUI. Figure 6 illustrates the GUI output for the query “colon cancer” on PubMed Central. The GUI has four main panels (see Figures 7–11) for exploring and analyzing the network (or BKG).

The central orange panel depicts the network nodes and edges. The nodes are labeled with their entities and colored by their biological categories (see the left panel in Figure 6 and Figure 7). The node size reflects its personalized PageRank. The user can select a graph layout from the drop-down menu at the bottom left of the central orange panel in Figure 6. The options are fCoSE[6], Spread[7], and Concentric[8].

The user can also download the network as SVG, CSV, or JSON files from the three buttons at the bottom of the central panel. The CSV files (nodes.csv and edges.csv) can be imported into graph databases or Cytoscape Desktop for further analysis or visualization. The JSON file contains all node and edge information (see the bottom-blue panel in Figure 6) and the sentences associated with each edge.

We now describe each panel of Figure 6, starting from the left panel in Figure 7. This panel has three sub-panels. The top one (highlighted in red) can be used to filter nodes and edges by two parameters: the network size (slider “Max items” from 50 to 300) and the edge weight (slider “Min weight” and “Bio coherence”). The middle one (highlighted in orange) can be used to select categories to display (or hide). The bottom one (highlighted in green) can be used to filter individual nodes by their labels.

When the user clicks on a node or an edge, the right (green) panel of Figure 6 shows its information in two drop-down menus: Node/edge details and Network analysis. For example, Figure 10 shows the information for the node “CCAT1” (green sub-panel in Figure 10a) and the edge “CCAT1 -> colon cancer” (orange sub-panel in Figure 10a), and the graph mining algorithms that can be applied to the network (see Figure 10b). The node “CCAT1” lists edges, their nodes, their labels (actions), and their PubMed sources. The edge colors in Figure 10a indicate the similarity (edit distance) between the edge label and a set of biological verbs, shown in Table 5 of the main paper. Green means a biological verb, which may interest the user; red means a non-biological verb, which may not. When the user clicks on an edge between the gene CCAT1 and the disease “colon cancer”, the right sub-panel “Edge details” shows its information, as in Figure 10a. The information includes the list of edges between these two nodes, their labels (actions), their weights (based on TF-IDF and the number of actions, see step 4 in Subsection 2.2 of the paper), their bio-coherence, and their PubMed sources. The table colors indicate the similarity (edit distance) between the edge label and a biological verb in Table 5 of the main paper. Two nodes can have multiple edges from different sentences. To avoid cluttering the network, we show one edge per node pair in the central orange panel in Figure 6 and its details in the Node/Edge-details panel. If the user clicks on an edge, a small window in the central panel displays the sentence that generated the edge, with the subject, object, and actions marked by their category colors (see Figure 8).

In addition, the user can suggest corrections or missing biological entities by clicking on the “Suggest corrections” button. An example is reported in Figure 9. Notice that updates are not automatically applied. Indeed, to maintain the quality of OntoTagMe, we will manually check such suggestions periodically and then rebuild the OntoTagMe database by adding the missing entities, thus generating a more exhaustive BKG.

The user can also apply graph-mining algorithms to the network by choosing them from the menu “Network analysis” in the right panel (see Figure 6 and Figure 10b). The algorithms are listed in four tabs: Traversal (for neighborhoods and connected components), Search (for BFS, DFS, and shortest paths), Centrality (for betweenness centrality and PageRank), and Clustering (for Markov, K-means or Hierarchical clustering).

The bottom panel of Figure 6 has four tabs (see Figure 11). The first tab, “Search data” (red box), lists the query parameters set by the user. The second tab, “Extracted articles” (orange box), shows the ID and title of the documents downloaded by NetMe 2.0 from PubMed/PubMed Central.

[4] https://fastapi.tiangolo.com/

[5] https://github.com/celery/celery

[6] https://github.com/iVis-at-Bilkent/cytoscape.js-fcose

[7] https://github.com/cytoscape/cytoscape.js-spread

[8] https://github.com/cytoscape/cytoscape.js-concentric-layout

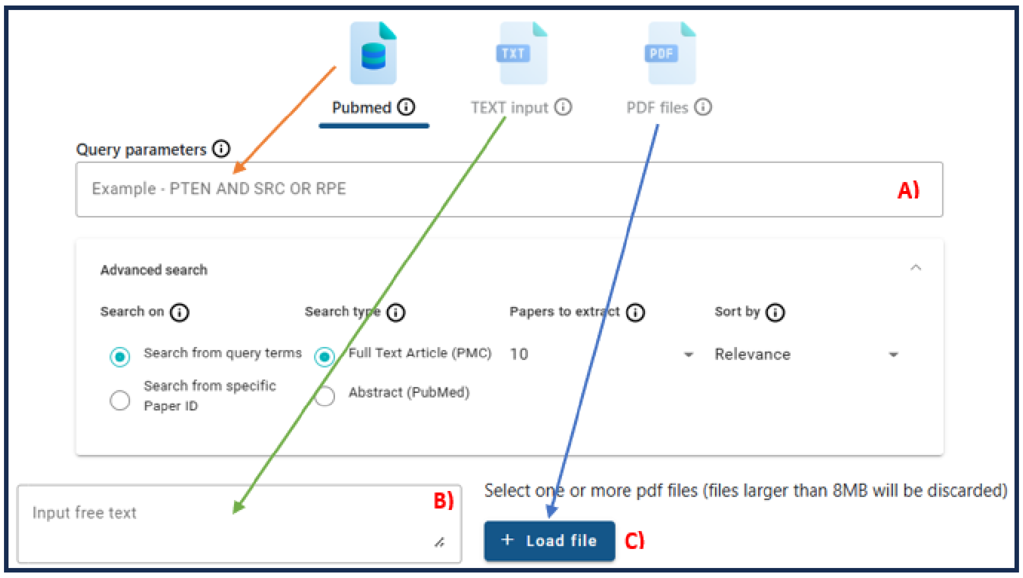

Fig. 5: The GUI panel to select the document sources for BKG construction: via (A) a query issued on PubMed; (B) a free text written within a textbox; or (C) a set of user-uploaded PDF documents

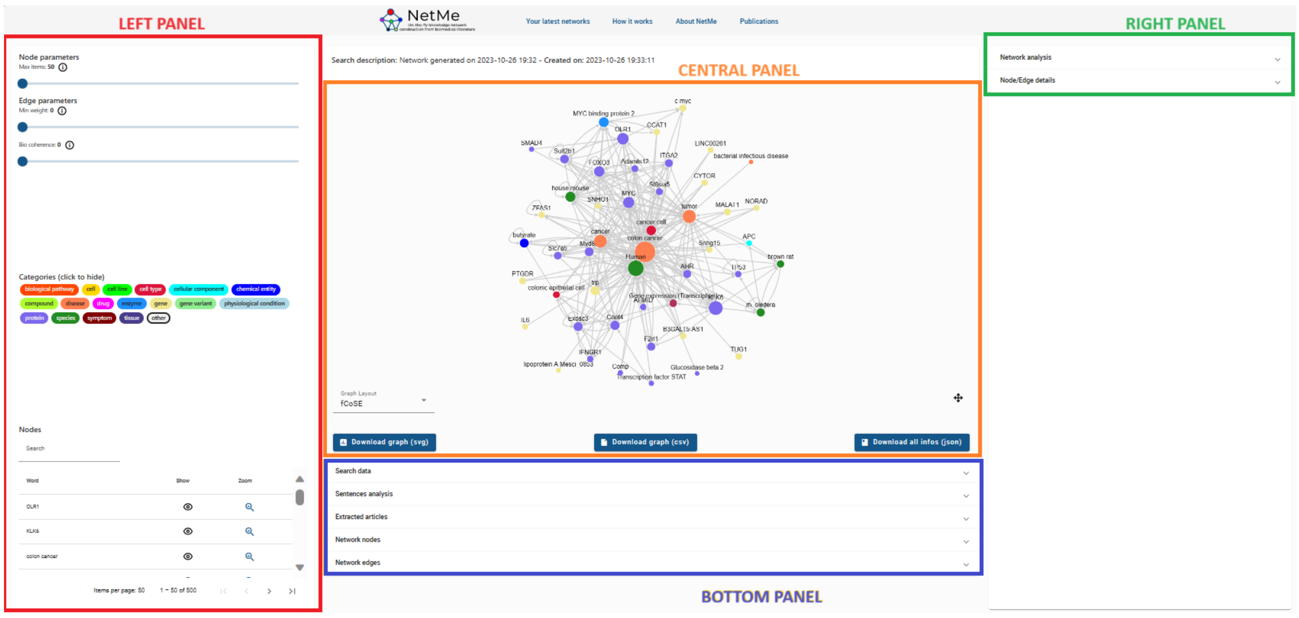

Fig. 6: NetMe 2.0 GUI showing the result of the query "colon cancer" posted on PubMed Central. The GUI consists of four panels that are boxed in red (left), orange (center), green (right), and BLUE (bottom), each one referring to a different feature that is commented on in the text

The last two tabs, "Network nodes" and "Network edges" (yellow and green boxes), show the node and edge information in tables.

2.4 Case Study 1 - Guided queries

We chose three genes {BRCA1, APP, BRAF} with many curated links in DisGeNET and various disease types. We filtered out associations that did not meet the following three criteria: (i) The *Evidence Level* must not be labeled as *limited* or *disputed*; (ii) The *GDA Score* (measuring literature evidence) must be above a given threshold (we used $0.3, 0.4, 0.5$); (iii) has at least 3 supporting PubMed IDs.

Next, for each GDA, we built a network on the supporting PubMed IDs. If NetMe found the GDA in their abstracts or open-access full-texts, we labeled it as "correct". Otherwise, we evaluated whether the missed edge was a "true miss" or not. Namely, we manually checked those abstracts and open-access full-texts, and if that GDA did not exist in them, we labeled that GDA as "fake miss"; otherwise, we labeled it as "true miss" because NetME should have found it.

Table 4 shows that NetMe 2.0 can find most of the curated edges in DisGeNET. Only 2 of 46 edges are *true misses* by NetMe (APP-Dementia and BRAF-LeopardSyndrome), while eight are missed due to the lack of evidence in the provided PubMed IDs.

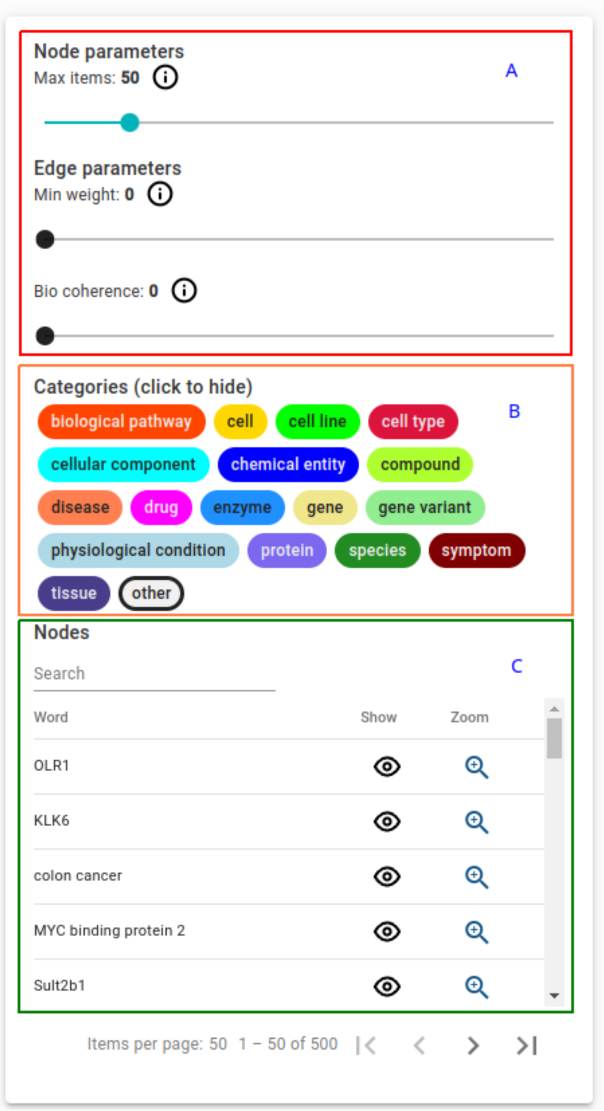

Fig. 7: Left panel of the GUI of NetMe 2.0

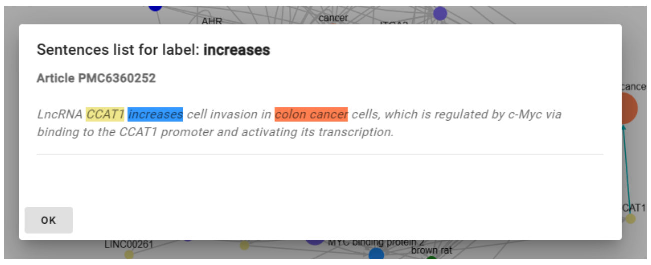

Fig. 8: Sentence obtained by clicking on the "increases" link under the "Edge" column of the Edge details table (orange box in Figure 10a). The string CCAT1 (subject of the sentence) is marked in yellow (because it belongs to the gene category), and colon cancer (object of the sentence) is marked in orange (because it belongs to the cancer category). In contrast, the action "increases" (verb of the sentence) is marked in blue

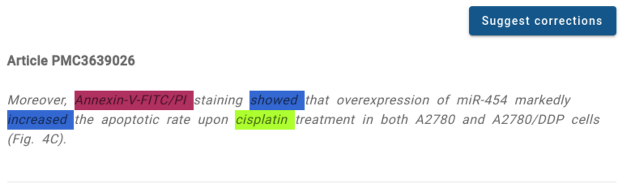

(a) "Suggest corrections" button

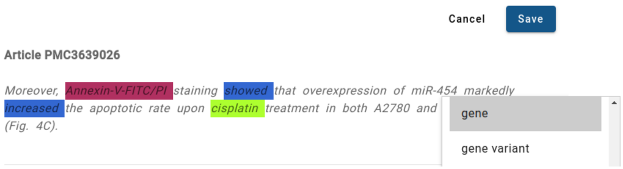

(b) Selection of "miR-454" and its own category (gene)

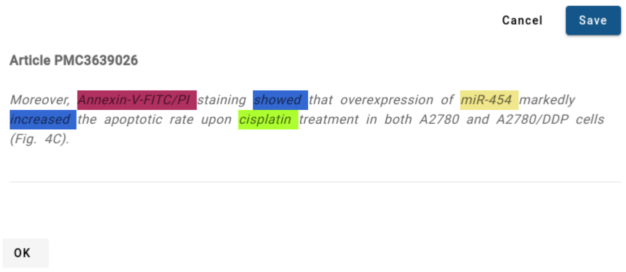

(c) "OK" button for sending every suggestion

Fig. 9: Example of missing biological term ("miR-454") suggestion by an user on the NetMe 2.0 GUI. When users find a missing/erroneous biological element, they should click on the "Suggest Corrections" button. Next, they can click on the desired term (i.e., "miR-454") and then select the appropriate biological category (gene in this example) through the drop-down menu. Finally, users can submit the suggestion by clicking the "OK" button, or alternatively, discard the changes by clicking on "Cancel".

Table 4. Case Study 1 - Metrics over three genes

Gene	Threshold	DisGeNET GDAs	NetME Correct	NetME True Misses	NetME Fake Misses
BRCA1	$S_{gda} \geq 0.3$	14	10	0	4
	$S_{gda} \geq 0.4$	5	5	0	0
	$S_{gda} \geq 0.5$	5	5	0	0
APP	$S_{gda} \geq 0.3$	21	16	1	4
	$S_{gda} \geq 0.4$	10	8	1	1
	$S_{gda} \geq 0.5$	6	4	1	1
BRAF	$S_{gda} \geq 0.3$	12	10	1	1
	$S_{gda} \geq 0.4$	9	8	1	0
	$S_{gda} \geq 0.5$	9	8	1	0

2.5 Case Study 2 - Free queries

For the second case study, we query NetMe using gene names, like in PubMed. We got all *DisGeNET curated GDAs* (updated to 2022). We chose two genes with more than 10 diseases, ABAT and CACNA1A. For each gene, we queried NetMe 2.0 to build a network with the top-50 most relevant full-text articles in PubMed Central, finding 72 GDAs for ABAT and 59 for CACNA1A. Next, we ranked them by NetMe edge weight, selected the top-20 GDAs, and then computed the accuracy.

Results (Table 5) highlight our ability to infer many significant GDAs without clear guidance on the paper selection for building the network.

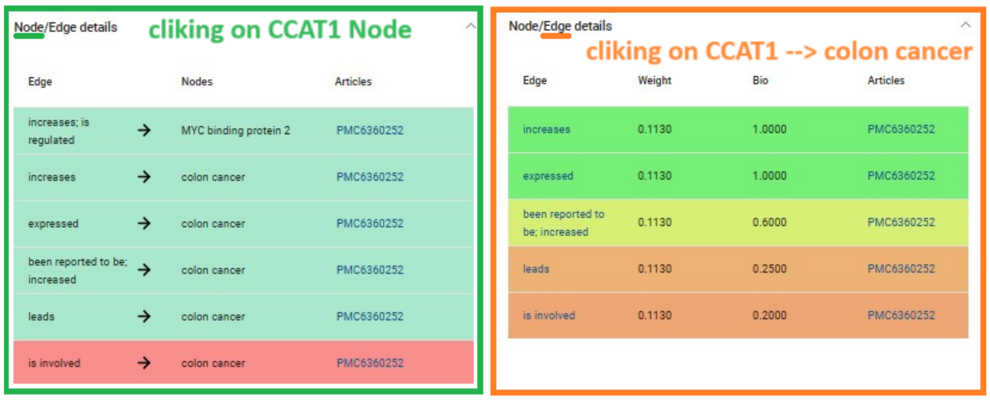

(a) Illustrative example of the information shown by the “Node/Edge details” tab located at the right (green) panel of the GUI

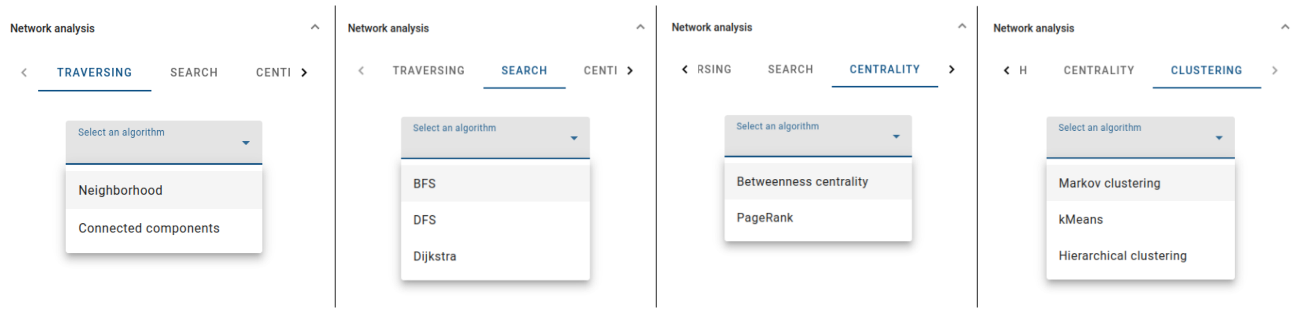

(b) Illustrative example of the information shown by the “Network analysis” tab located at the right (green) panel of the GUI

Fig. 10: This figure illustrates the information shown by the “Node/edge details” tab (top) and the “Network analysis” tab (bottom) that are located on the right side of the NetMe 2.0 GUI. An explanation of the picture is in the text

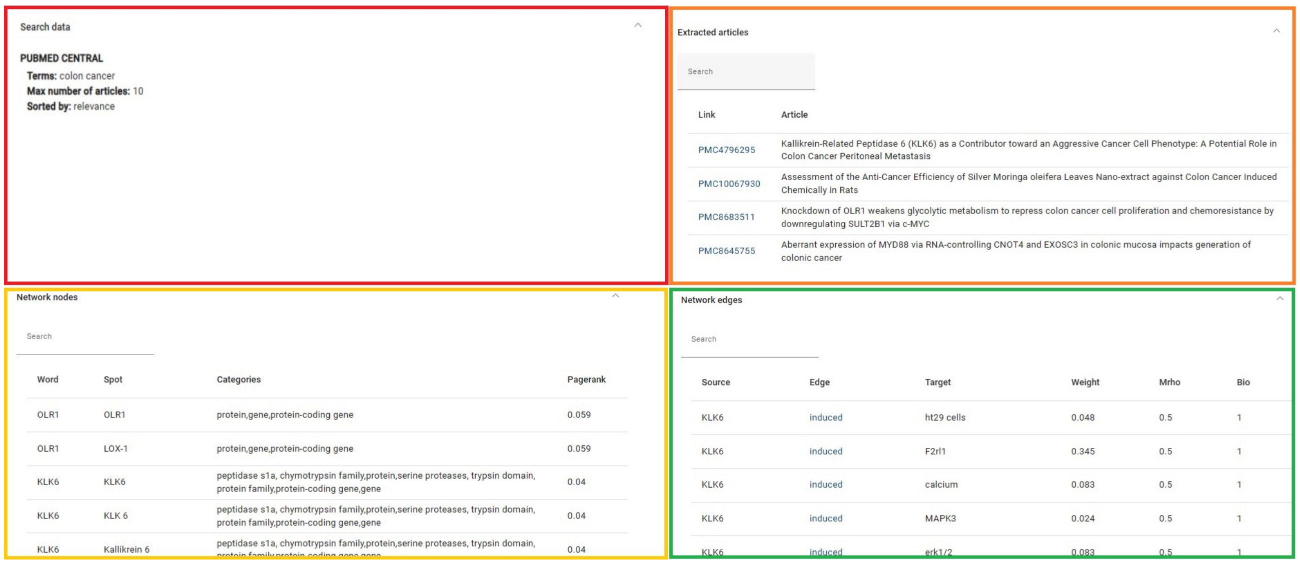

Fig. 11: Bottom panel of the NetMe 2.0 GUI

This is a crucial feature of NetMe that has been specifically developed to deal with the ever-growing amount of publications in PubMed and PubMed Central.

For this case study, we tested three tools. Namely NetMe, DARLING, and BioTagMe. We limited the comparison to them because they are the only ones providing a weight associated with each edge, and thus allow us to rank the results and compute the metrics specified below:

- *Found Edges*: Number of GDAs (edges) found by the tool due to a gene query.
- *Correct*: Number of correctly identified GDAs (edges) by the tool, with respect to the ones present in DisGeNET.
- *Correct@20*: Like “Correct”, but keeping only the top-20 GDAs found by the tool according to their (edge) weight.

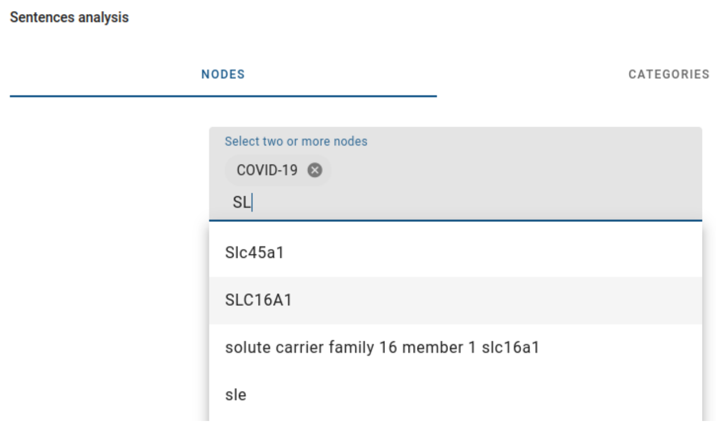

Fig. 12: The GUI panel to select the nodes — i.e., COVID-19 and SLC16A1 — which are the subject of the Graph-RAG invocation

Table 5. Case Study 2 - Metrics over two genes. For each metric, we bolded the best entries for the two test genes

Tool	Gene	DisGeNET GDAs	Found edges	Correct	Correct@20	Precision@all	Precision@20	Recall	Recall@20	F_1@20
NetMe 2.0	ABAT	13	72	10	9	0.14	**0.45**	**0.77**	**0.69**	**0.54**
	CACNA1A	12	31	7	5	**0.26**	**0.25**	0.58	**0.42**	**0.31**
DARLING	ABAT	13	4	3	3	**0.75**	0.15	0.31	0.23	0.18
	CACNA1A	12	71	6	3	0.04	0.15	0.5	0.25	0.19
BioTagMe	ABAT	13	187	8	0	0.02	0	0.62	0	0
	CACNA1A	12	546	10	0	0.04	0	**0.83**	0	0

- *Precision@all*: it mimics the behavior of the “*Precision*” metric (i.e., $P = {TP}/{TP+FP}$), specialized on this specific task: $P_{@all} = {\text{Correct}}/{\text{Found Edges}}$.
- *Precision@20*: Similar to Precision@all, but considering the top-20 edges only. It is computed as $P_{@20} = {\text{Correct@20}}/{20}$.
- *Recall*: Computed as $R = {\text{Correct}}/{\text{DisGeNET GDAs}}$.
- *Recall@20*: Computed as $R_{@20} = {\text{Correct@20}}/{\text{DisGeNET GDAs}}$.
- F_1*@20*: Computed as the classic F_1-metric, but replacing Precision and Recall with their values @20.

Notice that the precision values may be misleading. This is because false positives may indeed not be real false positives due to the incompleteness of DisGeNET as a gold standard.

Regarding NetMe's results, we manually checked that for the gene ABAT, NetMe correctly identifies 10 out of 13 GDAs (edges) of DisGeNET, with only one out of the 10 correct ones being out of the top 20 most relevant ones. We then zoomed on all these 3 misses (i.e., liver cirrhosis, developmental delay, convulsions) by looking at the abstracts and full texts in PubMed and PubMed Central where those diseases and the gene ABAT co-occurred. We found that none of them co-occurred in abstracts, but they co-occurred in some full texts in different paragraphs, thus making it impossible for NetMe to retrieve such a relationship. In detail,

- *ABAT - liver cirrhosis*: co-occurred in 6 full-text papers and never in abstracts.
- *ABAT - developmental delay*: co-occurred in 3 full-text papers and never in abstracts.
- *ABAT - convulsions*: co-occurred in 10 full-text papers and never in abstracts.

For the gene CACNA1A, NetMe found 7 out of 12 edges, and 2 of the correct ones were not in the top 20 results. The 5 missed edges had some form of evidence on PubMed, as indicated by DisgeNET. But, since NetMe relies on the PubMed search engine, it has no guarantee to retrieve the *good* papers for CACNA1A.

2.6 Case Study 3 - BKG comparison on authoritative edges

In this Case Study, we run the same experiment as the Case Study presented in section 3.1 of the main paper, but focusing on high-quality GDAs from DisGeNET while varying the source genes. More specifically, we took the list of GDAs present in DisGeNET and grouped them by genes. Then we dropped all the GDAs that:

- had a $\mathbf{Score}_{\text{GDA}} \leq 0.5$;
- were not in the manually curated set;
- had an $\mathbf{EL}_{\text{GDA}}$ either “limited” or “disputed”;
- had up to 2 supporting PubMed IDs.

This filtering resulted in 64 authoritative GDAs referring to 20 different genes (i.e., genes TGFB1, IL10, KRAS, TLR2, ACE, BDNF, PTGS2, ESR1, APOE, EGFR, IL1B, TP63, PTEN, CDKN2A, CRP, BCL2, TP53, IL2, ERBB2, and MMP2), over which we ran the experiment discussed section 3.1 of the main paper by querying those genes, and finally obtained the figures shown in the following Table 6. Our tool clearly demonstrates its superiority over other state-of-the-art approaches in constructing BKGs.

References

Carvalho-Silva, D. *et al.* (2018). Open Targets Platform: new developments and updates two years on. *Nucleic Acids Research*, **47**(D1), D1056–D1065.

Krallinger, M. *et al.* (2017). Overview of the biocreative vi chemical-protein interaction track. In *Proceedings of the sixth BioCreative challenge evaluation workshop*, volume 1, pages 141–146.

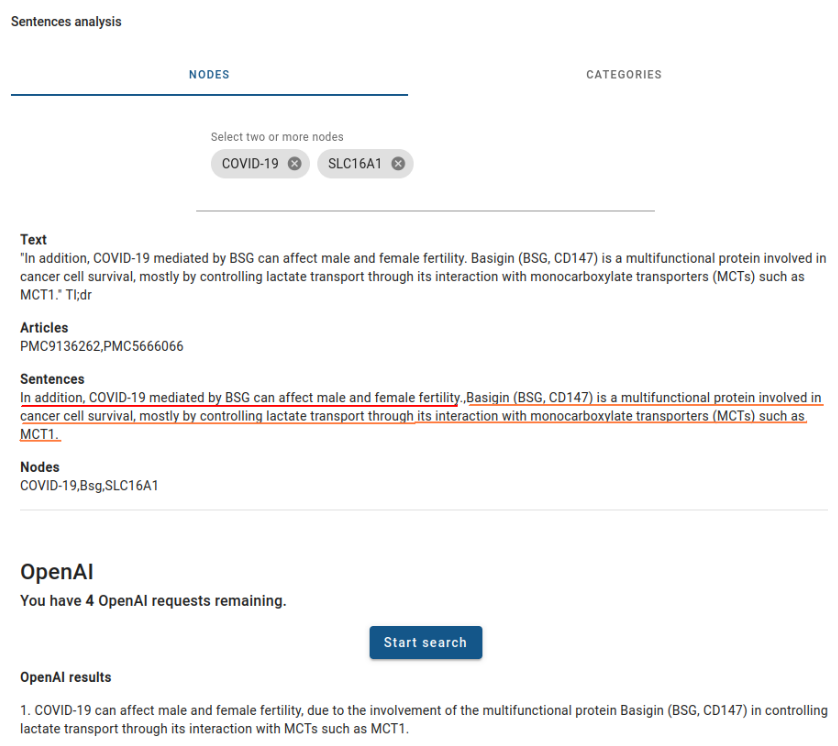

Fig. 13: An example of interaction with the Graph-RAG module. The user selects two or more nodes, as indicated in Figure 12: i.e., "COVID-19" and "SLC16A1". The system retrieves a set of sentences labeling the connecting paths (i.e., two in the picture), listed within the "Sentences" section and underlined in red and orange. Furthermore, in the "Articles" section, the GUI shows the document IDs from which sentences were extracted, and in the "Nodes" section, the nodes involved in the connecting paths. When the "Start search" button is clicked, the sentences are sent to OpenAI, which will return a summarized text. Each KG allows users to submit no more than five requests to OpenAI. If more requests are needed, the button "Start search" is automatically replaced by the GUI with a text box where users can input their API key. Such a key is not sent to our backend, as required by OpenAI developers

Table 6. Comparison with other BKGs - accuracy on 64 authoritative GDAs

Graph type	Tool	Correct	Edges extracted from	web-app
built on-the-fly with labeled and weighted edges	**NetMe 2.0 (this paper)**	**62**	full-texts and abstracts	yes
precomputed with labeled and weighted edges	BIOS	0	abstract	yes
	BioKG	43	ontologies	no
	SPOKE	39	ontologies	yes
	Hetionet	28	ontologies	yes
precomputed with weighted edges	Darling	36	abstract	yes
	BioTagMe	53	abstract and ontologies	yes

Krallinger, M. *et al.* (2020). Drugprot shared task (biocreative vii track 1-2021) text mining drug-protein/gene interactions (drugprot) shared task.

Szklarczyk, D. *et al.* (2018). STRING v11: protein–protein association networks with increased coverage, supporting functional discovery in genome-wide experimental datasets. *Nucleic Acids Research*, **47**(D1), D607–D613.

Zitnik, M. *et al.* (2018). Modeling polypharmacy side effects with graph convolutional networks. *Bioinformatics*, **34**(13), i457–i466.
